# Analysis of Sporulation in *Bacillus cereus* Biovar *anthracis* Which Contains an Insertion in the Gene for the Sporulation Factor σ^K^

**DOI:** 10.3390/pathogens12121442

**Published:** 2023-12-13

**Authors:** Constanze Gummelt, Susann Dupke, Sabine Howaldt, Fee Zimmermann, Holger C. Scholz, Michael Laue, Silke R. Klee

**Affiliations:** 1 Highly Pathogenic Microorganisms (ZBS 2), Centre for Biological Threats and Special Pathogens, Robert Koch Institute, 13353 Berlin, Germany; gummeltc@rki.de (C.G.); dupkes@rki.de (S.D.); howaldts@rki.de (S.H.); scholzh@rki.de (H.C.S.); 2 Epidemiology of Highly Pathogenic Microorganisms (P3), Robert Koch Institute, 13353 Berlin, Germany; fee.zimmermann@helmholtz-hioh.de; 3 Advanced Light and Electron Microscopy (ZBS 4), Centre for Biological Threats and Special Pathogens, Robert Koch Institute, 13353 Berlin, Germany; lauem@rki.de

**Keywords:** *Bacillus cereus* group, sigma factor, site-specific recombination, gene expression analysis, electron microscopy, ultrastructure

## Abstract

*Bacillus cereus* biovar *anthracis* (*Bcbva*) is an untypical pathogen causing a fatal anthrax-like disease in a variety of wildlife species in African rainforest areas. In contrast to *Bacillus anthracis* and most species of the *B. cereus* group, all strains of the *Bcbva* cluster contain a 22 kb insertion in the *sigK* gene which encodes the essential late sporulation sigma factor σ^K^. This insertion is excised during sporulation in a site-specific recombination process resulting in an intact *sigK* gene and a circular molecule. The sporulation kinetics of two strains each of *Bcbva* and *B. anthracis* were compared by the expression analysis of eight sporulation-associated genes, including *sigK*, using reverse transcriptase quantitative real-time PCR. In addition, morphological sporulation stages were analyzed and quantified by electron microscopy. Our results indicated that the necessary excision of the insertion in *Bcbva* neither delayed nor inhibited its sporulation. In two spontaneous mutants of *Bcbva*, the excision of the *sigK* insertion and sporulation were impeded due to mutations in the *spo0A* and *spoVG* regulator genes, respectively. The *spo0A* frameshift mutation was overcome by intragenic suppression in a revertant which was able to sporulate normally, despite an M171S amino acid exchange in the global regulator Spo0A. A screening of the NCBI database identified further strains of the *B. cereus* group which possess unrelated insertions in the *sigK* gene, and two strains containing almost identical insertions at the same gene position. Some of the *sigK* insertions encode putative prophages, whereas the *Bcbva* insertion encoded a type I restriction–modification system. The function of these insertions and if they are possibly essential for sporulation remains to be assessed.

## 1. Introduction

Anthrax, caused by *Bacillus anthracis* (*Ba*), is a worldwide-occurring zoonotic disease primarily affecting herbivores in arid regions like the savannas in sub-Saharan Africa. *Bacillus cereus* biovar *anthracis* (*Bcbva*) can also cause an anthrax-like disease, but is untypical due to several features. To date, it has only been detected in African rain forest areas where it caused anthrax-like symptoms in a large number of wild animals of different species, including chimpanzees, gorillas, elephants, different monkeys and duikers [1,2,3]. Based on a *B. cereus*-like chromosomal background, *Bcbva* possesses both virulence plasmids pXO1 and pXO2 of *Ba* and consequently produces anthrax toxins and capsules. The similar virulences of *Bcbva* and classic *Ba* were shown in small animal models [4], and human exposure to *Bcbva* was confirmed by seroprevalence studies [5]. Phylogenomic analyses revealed that *Bcbva* belongs to a discrete clade in the *B. cereus* group separate from the monophyletic *B. anthracis* clade [6]. *Bcbva* strains are lacking the four *Ba*-specific prophage regions, but their genomes contain five to six genomic islands which can be used for the specific identification of this agent [7]. As members of the genus *Bacillus*, both *Ba* and *Bcbva* have the ability to form endospores, which are metabolically inactive and resistant to heat, chemicals, UV radiation or desiccation. They are formed within the bacterial cell by a complex morphogenesis termed sporulation whenever the vegetative cell is exposed to physiological stress, such as nutrient limitation or high cell density [8]. By forming spores, the bacteria manage to survive in dormancy even for decades before recovering to the vegetative form by germination when environmental conditions are suitable [9]. Due to their extreme environmental resistance, spores play an important role in bacterial dispersal. In strains relevant to human and veterinary health, such as *B. anthracis*, spores are mainly responsible for infection [10]. Moreover, spores of *B. anthracis* have been used as bioweapons in the past, because they can easily be produced in high amounts and efficiently distributed [11].

*Bacillus subtilis* is the standard model for the study of spore development and structure [12]. The spore core is limited by a biomembrane with a thin outer layer of peptidoglycan (i.e., the primary cell wall) and contains the bacterial chromosome which is tightly packed with small acid-soluble proteins (Sasp) in a crystalline-like form to protect the DNA [13]. The core is surrounded by the cortex, the inner and the outer spore coats. During sporulation, the core water is drastically reduced which is one reason for the extraordinary resistance of the spores. The two spore coats are mainly composed of proteins and provide a barrier against larger chemical agents or enzymatic degradation [8]. However, the main barrier against chemicals seems to be the biomembrane of the core which does not show any lipid mobility (see [14] for a detailed review of the resistance mechanisms of spores). The bacteria of the *B. cereus* group possess an additional layer around the coats, termed exosporium, with which they are loosely connected. The surface of the exosporium is covered by a hair-like nap mainly composed of the collagen-like glycoprotein BclA [15]. The function of the exosporium is not yet conclusively understood, but it appears to play a role in interaction with the environment [16].

Under growth-limiting conditions, the sporulation process is initiated in vegetative cells. The metabolism is minimized during sporulation, and the timing for the expression of the mRNAs responsible for spore development is coordinated by a cascade of transcription factors (σ factors). The regulation of sporulation in *B. subtilis* has been summarized in several reviews [8,17,18,19] and seems to be similar in *B. anthracis* and the *B. cereus* group, although some exceptions were observed [20,21,22]. The transition from vegetative growth to sporulation is essentially determined by the global regulator Spo0A, a DNA binding protein. The activation of histidine kinases, which respond to different kinds of stresses, leads to phosphorylation of this major transcription factor, whose phosphorylation state controls its activity and is relevant for the regulation of gene expression [18]. During the first stage of sporulation, the chromosome duplicates. In the later stages, it is distributed between the mother cell and the so-called forespore. This morphological differentiation results from the asymmetric division of the mother cell with the initially centric septum shifting to one of the cell poles. This transformation into a polar septum is dependent on the production of the SpoIIE membrane phosphatase, which is also involved in the activation of σ^F^ within the forespore [17,18]. The activity of σ^F^ in the forespore triggers the activation of σ^E^ in the mother cell. The transcription of the σ^E^-regulon in the mother cell activates the gene for the regulatory protein SpoIIID and leads to the expression of the proteins required for engulfment. During this step, the mother cell completely encloses the forespore by forming a double membrane. A zipper mechanism separates the mother cell from the forespore by detaching the membranes so that the forespore is exposed to the cytosol of the mother cell at the end of engulfment [8]. Once the engulfment is completed, σ^G^ is activated in the forespore, which in turn activates σ^K^ in the mother cell. Products of the σ^K^-controlled genes synthesize the cortex (e.g., *murF*), small acid soluble proteins (e.g., *sasp1*), and assemble the spore coat (e.g., *cotZ*), before the mature spore is released at the stage of mother cell lysis [17]. Strikingly, in the mother cell, many genes activated by a particular transcription factor are again inhibited by the next regulator in the cascade [17]. In *B. subtilis*, the pleiotropic regulator SpoVG negatively regulates asymmetric septum formation at an early stage of sporulation, whereas it positively regulates cortex formation at a later stage [23]. Mutants of *spoVG* only have minor defects in sporulation due to redundant factors. In *B. anthracis*, however, the disruption of *spoVG* resulted in defective sporulation due to the mutants’ inability to form an asymmetric septum [22].

A key position in the sporulation of *B. subtilis* is occupied by the sigma factor σ^K^, which activates about 100 genes. A 48 kb so-called *skin* element is inserted in the *sigK* gene [24], seemingly a phage-like element with the majority of its genes encoding phage proteins. Before the gene is transcribed, the *skin* element is excised by a reciprocal site-specific recombination process in the region of an inverted repeat [25], within which the coding regions of the *sigK* gene are linked, and the insertion is removed from the chromosome in the form of a circular molecule [26]. The recombinase SpoIVCA, responsible for this process, is also encoded on the *skin* element. Since the recombination process only occurs in the mother cell, the spore receives a *sigK* gene in which the *skin* element is still present. The regulator SpoIIID activates the transcription of the *spoIVCA* and *sigK* genes [27], resulting in a newly formed σ^K^ precursor protein. Subsequently, the as-yet inactive pro-σ^K^ is activated by the membrane-bound protease SpoIVFB, which is also complexly regulated and activated by, among others, σ^G^ in the forespore [8]. For the *B. cereus* group, insertions in the *sigK* gene were not known before the sequence of *Bcbva* CI was published [7]. The 22 kb genomic island IV is inserted in the *sigK* gene and apparently excised during sporulation. In order to examine if the insertion exerts any effect on the sporulation capacity of *Bcbva*, a strain which is able to sporulate efficiently, we compared the sporulation kinetics and regulation of selected genes between classical *Ba* and *Bcbva* in two strains of each. This study was complemented by electron microscopy showing the different sporulation stages. We also analyzed two spontaneous sporulation mutants of *Bcbva* which were unable to excise the insertion. Finally, we screened the NCBI database to detect further *B. cereus* group strains possessing an insertion in the *sigK* gene.

## 2. Materials and Methods

### 2.1. Strains, Growth Conditions and Determination of Sporulation Efficiency

*B. anthracis* Vollum (A.Br.Vollum; ATCC 14578, type strain; GenBank accession number NZ_CP076225) is a well-known laboratory strain, whereas *B. anthracis* 14RA5914 (A.Br.001/002; GenBank accession number NZ_CP023001) was only recently isolated from a cow in Germany [28]. The *B. cereus* biovar *anthracis* strains CI (GenBank accession number CP001746) and CA (NCBI BioSample SAMN03610234) were isolated from chimpanzees in Côte d’Ivoire and Cameroon [6,29], and the spontaneous mutants CI-12 and CA-2 were derived from these strains after repeated subcultivation and selection of aberrant phenotypes. Strains were routinely cultured in Luria Bertani (LB) medium, and sporulation was induced by diluting an overnight culture in 20 mL of modified Medium G (MGM, [30]) to an optical density (OD_600_) of 0.03–0.05. Three separate cultures per strain were grown while shaking (200 rpm) at 37 °C, and the OD_600_ was measured each hour to determine the initiation of sporulation (t_0_), which is defined by the end of exponential growth [22,31,32].

To detect revertants of the sporulation mutants CA-2 and CI-12, three cultures of 20 mL of each mutant were grown in MGM at 37 °C for eight days. The cultures were 10-fold concentrated by centrifuging (5000× *g*, 20 min) and resuspending in 2 mL of saline, to allow detection of minor amounts of spores. The concentrated cultures were heat-inactivated (65 °C, 30 min) to kill vegetative cells; 100 µL was plated on LB agar (direct culture), and 500 µL was inoculated into 10 mL of LB (enrichment culture).

To compare sporulation efficiency of wild type *Bcbva* CI and of the CI-12 revertant, three separate cultures were each grown in MGM at 37 °C for 24 h with shaking. The fraction of heat-resistant spores was determined by calculating the colony-forming units (cfu) of heat-treated (65 °C, 30 min) and untreated cultures.

### 2.2. PCR and Sequencing

Supernatants of heated (95 °C, 30 min) and centrifuged bacterial cultures were used as crude DNA for PCR and subsequent Sanger sequencing under standard conditions. Whole genome sequencing of mutants *Bcbva* CA-2 and CI-12 was performed, as previously described [3]. All primer sequences are listed in Supplementary Table S1.

### 2.3. Southern Blot

DNA from cultures grown in MGM for two, four and six hours, as described above, was isolated using the DNeasy Blood & Tissue Kit (Qiagen, Hilden, Germany) and separated on a 0.7% agarose gel. PCR fragments were generated with primers sigK-3 and sigK-4 and labeled by PCR DIG labeling mix (Roche, Mannheim, Germany). Southern analysis was performed as described earlier [29].

### 2.4. RNA Isolation, cDNA Synthesis and Reverse Transcription Real-Time Quantitative PCR

From each strain, three separate cultures were grown in MGM, as described above, and 3 mL aliquots were centrifuged at time points t_0_ (start of sporulation), one hour (t_1_) and two hours later (t_2_). RNA extracted from bacterial pellets was treated by DNase I, as described earlier [4]. A Qubit fluorometer (Thermo Fisher Scientific, Waltham, MA, USA) served to determine RNA concentration. For synthesis of cDNA, the SuperScript™ First-Strand Synthesis System (Invitrogen, Karlsruhe, Germany) was used with 30 ng of RNA and 1 µL (50 ng) of random hexamer primers in 20 µL reactions. Primers for amplification of selected genes (amplicon sizes 80–160 bp) were designed using the GenScript (URL www.genscript.com (assessed on 6 November 2023)) Online PCR Primers Designs Tool and are listed in Supplementary Table S1. The primer sequences were verified to be 100% identical to the sequences of all four strains tested. RT-qPCR reactions were performed on an ABI7500 Real-Time PCR System (Applied Biosystems, Darmstadt, Germany) in a final volume of 25 µL using 1 µL of cDNA, 12.5 µL of SYBR^®^ Green I PCR Master Mix (Applied Biosystems/Life Technologies, Darmstadt, Germany) and 300 nM of the appropriate forward and reverse primers. Cycling conditions were 95 °C for 10 min, followed by 40 cycles at 95 °C for 15 s and 60 °C for 30 s. A melting curve analysis resulted in individual product-specific melting temperatures for all samples. Negative control reactions using RNA not treated with reverse transcriptase were also included. PCR efficiencies for each primer combination were calculated by the ABI7500 software after measuring tenfold dilutions of genomic DNA of each strain.

For analysis of gene expression, the ΔCq method was applied using a reference gene (modified from [33]). To obtain a normalized expression value, expression of the variably regulated target genes (sporulation genes indicated in Table 1) was normalized against a non-regulated reference gene. We used the *rpoB* gene which is stably expressed throughout the bacterial life cycle [34,35]. First, the Cq values for the three technical replicates gained from each sample were averaged. For each target gene in each of the biological replicates and time points, the gene expression was normalized to the geometric mean of the Cq values obtained for the reference gene *rpoB* (∆Cq value) and transformed into linear scale expression quantities using the formula 2^−∆Cq^. Averages and standard errors were then calculated from the normalized expression values of the three biological replicates using the GraphPad Prism 9 Software.

### 2.5. Electron Microscopy

Strains were grown in MGM as described above, and culture aliquots were pelleted (at 1500× *g*, 15 min) after 2 h, 4 h, 5 h, 6 h, 7 h, 8 h, and 9 h. For the two sporulation mutants *Bcbva* CI-12 and CA-2, samples were only taken after 2 h and 24 h. Pellets were resuspended in 250 µL of 2.5% glutaraldehyde in 0.05 M HEPES buffer and fixed overnight. To ensure complete inactivation of spores, cells were pelleted again, resuspended in 250 µL of 20% formaldehyde/0.1% glutaraldehyde and incubated overnight. For electron microscopy, pellets of inactivated cells were embedded in low-melting point agarose (resuspension of the pellet in a double volume of agarose), and excised agarose blocks were treated with 1% OsO_4_ and 2% uranyl acetate. After stepwise dehydration with increasing ethanol concentration and embedding in LR White Resin (hard grade, Science Services, Munich, Germany), samples were sectioned by an ultramicrotome (UC7, Leica Microsystems, Wetzlar, Germany) and counter-stained with uranyl acetate and lead citrate. A transmission electron microscope operated at 120 kV (Tecnai12 Biotwin, Thermo Fisher, Eindhoven, The Netherlands) and a CCD camera (Megaview III, EMSIS, Münster, Germany) were used for analysis and documentation. To quantify the frequency of the different sporulation stages, representative sections were taken from samples fixed at different points of sporulation to count the number of cells at each stage.

## 3. Results

### 3.1. Confirmation of a sigK Insertion in Bcbva and Its Excision during Sporulation

**Figure 1:**
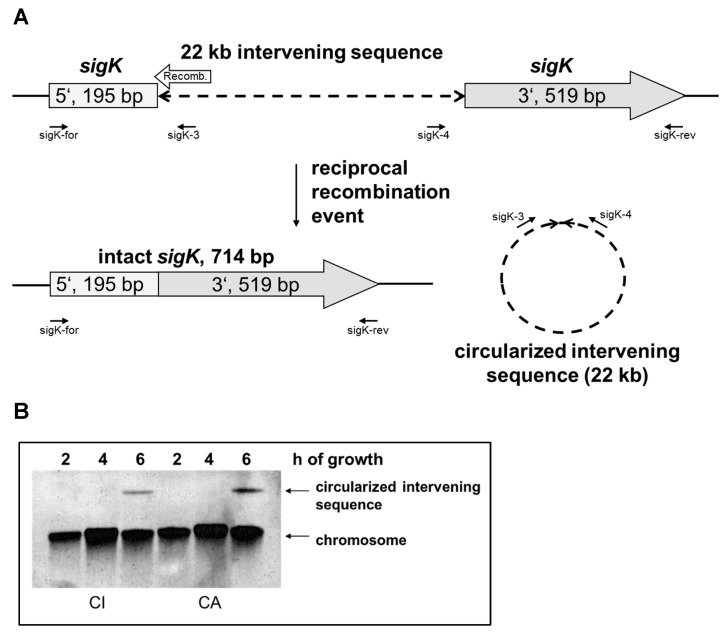
(**A**) Insertion of island IV in *sigK* gene and products of recombination event. The location of the recombinase gene (Recomb.) and relevant primers is shown (not drawn to scale). (**B**) Southern blot with DNA from *Bcbva* CI and CA cultures grown for 2, 4, and 6 h, respectively. Bands corresponding to chromosomal DNA and the circularized insertion are indicated.

Six genomic islands were identified in the genome of the *Bcbva* strain CI [7]. The largest one, denominated “island IV”, has a size of 22,211 base pairs and is inserted into the *sigK* gene at position 195 (from start codon). In contrast to *B. subtilis*, where many proteins encoded by the *skin* insertion have homologies to prophage proteins, no evidence could be found for a prophage insertion in *Bcbva*. Besides the hypothetical proteins, the 17 proteins encode a putative type I restriction–modification system and other proteins involved in DNA metabolism, like recombinases, endonucleases, and proteins with a helix-turn-helix domain (Supplementary Table S2). Adjacent to the 5′ fragment of the *sigK* gene, but in the opposite direction, a gene for a putative recombinase (BACI_c43240) is located, which is assumed to catalyze the excision, as the recombinase gene of *B. subtilis* (*spoIVCA*) is located similarly. In both species, the open reading frames of the *sigK* 5′ fragment and the recombinase are slightly overlapping (Figure 1A). The recombinase proteins SpoIVCA of *B. subtilis* (500 aa) and *Bcbva* CI (545 aa) are 31% identical (54% positives). The recombination site in *Bcbva* is flanked by 16 bp imperfectly inverted repeats and 18 bp imperfectly repeated sequences within which the recombination takes place (Supplementary Figures S1 and S5B). In *B. subtilis*, the site of recombination is characterized by the sequence AATGA which was not identified in *Bcbva* (Supplementary Figure S5B). As the site-specific recombination event only takes place in the mother cell, the spore retains a chromosomal copy with the insertion.

To confirm the reciprocal recombination event, overnight cultures of *Bcbva* strains CI and CA were diluted in sporulation medium (MGM), and DNA was extracted after two, four and six hours of growth. As shown by Southern blot analysis, a molecule corresponding to the circularized intervening sequence was visible only after six hours of growth (Figure 1B). The probe was generated by PCR with primers sigK-3 and sigK-4, which are able only to amplify the circularized, excised molecule (Figure 1A). However, as these sequences are also present in the chromosome, signals linked to the chromosomal bands were visible at all time points. Sequencing of the sigK-3/sigK-4 PCR product resulted in the sequence predicted from the excision event. Presence of the intact *sigK* gene after six hours of growth was confirmed by PCR and sequencing with primer pair sigK-for and sigK-rev (Supplementary Figure S1). Primer sequences are provided in Supplementary Table S1. The position of the insertion and the sequences surrounding the recombination site are identical in *Bcbva* strains CI (Côte d’Ivoire), CA (Cameroon), RCA (Central African Republic) and DRC (Democratic Republic of Congo). Not more than three nucleotide differences (point mutations or one nucleotide deletion) were observed in the insertion sequences of the four strains, which did not, however, affect the open reading frames. The sequences of the intact *sigK* genes of these four *Bcbva* strains are not only identical to each other but also 100% and 99.3% (5 mismatches) identical to the genes of the closely related strains *B. thuringiensis* serovar konkukian 97–27 and *B. anthracis* Ames, respectively.

### 3.2. Expression Patterns of Sporulation-Associated Genes

The insertion in the *sigK* gene of *Bcbva* might influence the regulation cascade of the sporulation-associated genes and the kinetics of sporulation itself. To substantiate this assumption, sporulation of the two *Bcbva* strains CI and CA was compared with the two *Ba* strains Vollum and 14RA5914. Sporulation is generally considered to start upon entry into the stationary growth phase. This point (t_0_) could be determined after analyzing the growth curves of the four strains, and was reached after four hours for *Bcbva* CI and CA and after five hours for *Ba* Vollum and 14RA5914 (Supplementary Figure S2). RNA was extracted for gene expression analysis at time points t_0_ and one (t_1_) and two (t_2_) hours later, corresponding to five and six hours for *Bcbva* CI and CA, and to six and seven hours for *Ba* Vollum and 14RA5914, respectively. The genes selected for expression analysis are listed in Table 1. These were chosen to cover different sporulation stages and regulatory or structural functions either executed in the mother cell or in the forespore. The constitutively expressed housekeeping gene *rpoB* was chosen for normalization.

**Table 1:** Sporulation-associated genes used for expression analysis.

Gene Name	Locus Tag ^a^	Function	Sporulation Stage	Wave ^b^ [20]	Wave ^b^ [21]
*sigF*	BA_4294	Sporulation-specific sigma factor σ^F^	Formation of asymmetric septum, F	2	3
*spoVG*	BA_0047	Sporulation-specific regulator	Formation of asymmetric septum, M	2	3
*spoIIID*	BA_5521	Sporulation-specific regulator	Start of engulfment, M	4	4
*sigG*	BA_4042	Sporulation-specific sigma factor σ^G^	End of engulfment, F	4	4
*sigK*	BA_4566	Sporulation-specific sigma factor σ^K^	End of engulfment/spore differentiation, M	5	4
*murF*	BA_0246	Protein of spore cortex	Formation of spore cortex, F	5	absent
*cotZ*	BA_1234	Protein of spore coat	Formation of spore coat, F	5	5
*sasP1*	BA_0858	DNA protecting small acid soluble protein	Spore differentiation, F	5	5

^a^ in *B. anthracis* strain Ames; ^b^ wave in which gene expression was observed in the studies by Liu et al. [20] or Bergman et al. [21]; M: active in mother cell; F: active in forespore.

**Figure 2:**
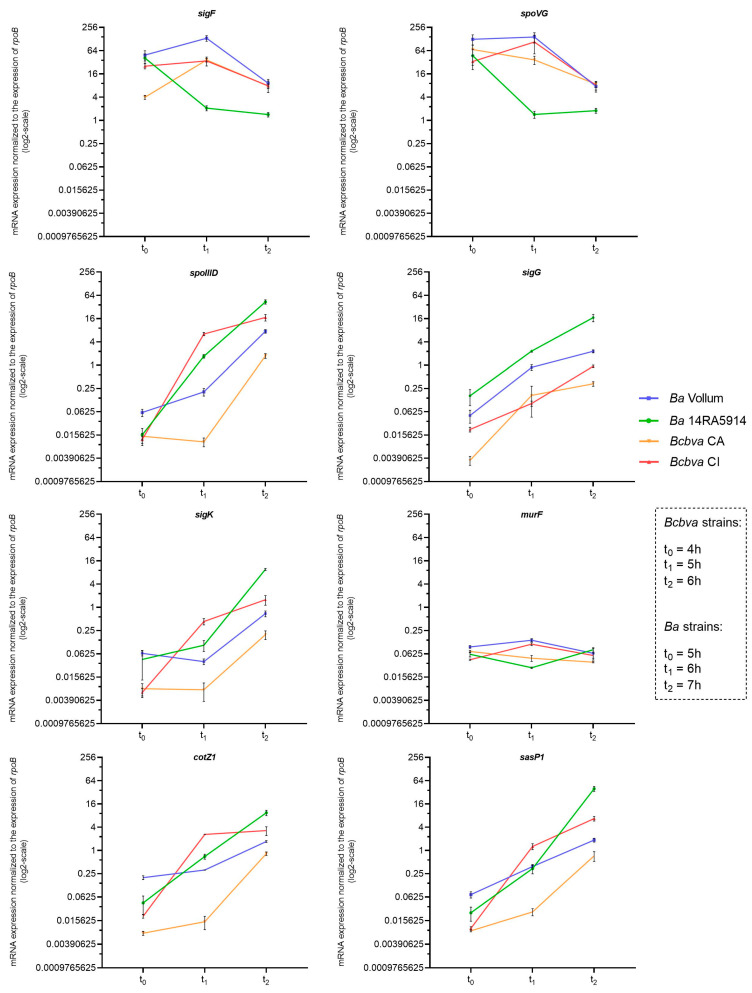
Reverse transcriptase (RT) qPCR-based expression profiles of sporulation-associated genes *sigF*, *spoVG*, *spoIIID*, *sigG*, *sigK*, *mur*, *cotZ1* and *sasP* in *Ba* and *Bcbva* strains. For each gene, levels of mRNA transcripts were determined by RNA sampling at time points t_0_, t_1_ and t_2_, corresponding to 5, 6, and 7 h of growth for *Ba* strains and 4, 5, and 6 h of growth for *Bcbva* strains. For each target gene, relative transcript expression levels were determined by normalization to the control gene *rpoB*. The mean and standard error values from three independent experiments are shown.

To allow a comparison of the different PCR assays, it was confirmed for all primer combinations that the variability of the efficiency did not exceed 10% (Supplementary Table S3). The expression profiles are presented in Figure 2. For *sigF* and *spoVG*, which are active at an early stage of spore development, expression was highest at time points t_0_ or t_1_ and decreased towards t_1_ and t_2_ for all strains, respectively. The expression of the downstream regulated genes *spoIIID*, *sigG*, *sigK*, *cotZ* and *sasP1* showed similar patterns between the two species with expression increasing over time showing a peak at t_2_. Over 3 h of examination, no remarkable changes in gene expression could be observed for *murF* in the *Ba* and *Bcbva* strains in question. The maximum differences in expression were a 42-fold increase in *spoIIID* and a 39-fold increase in *sasP1* expression in the *Ba* 14RA5914 strain between t_0_ and t_2_. Altogether, the highest mRNA expression values were observed for *sigF* (value of 134) and *spoVG* (value of 144) in the *Ba* Vollum strain at time point t_1_. Compared to the other strains, *Bcbva* CA displayed the lowest expression levels for five of the eight investigated genes (*spoIIID*, *sigG*, *sigK*, *cotZ* and *sasP1*).

### 3.3. Analysis of Sporulation Stages by Electron Microscopy

**Figure 3:**
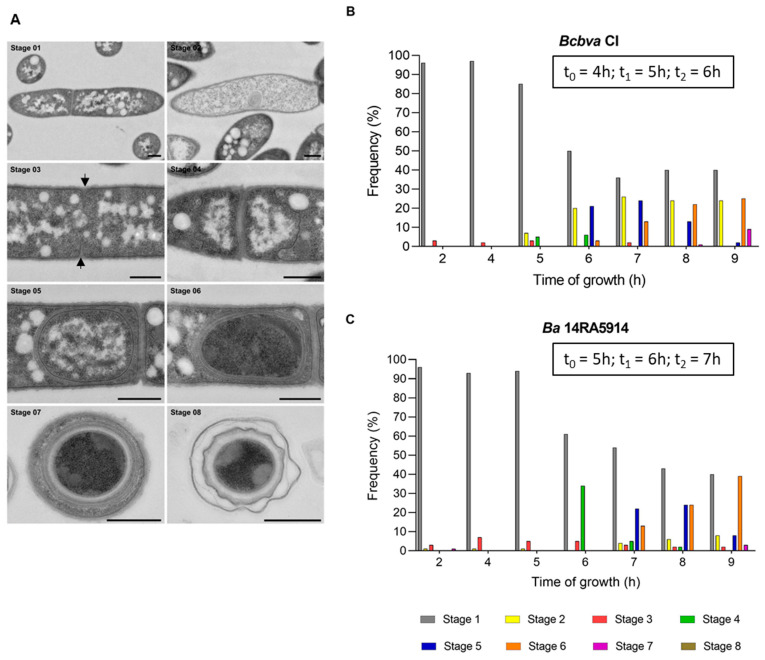
Sporulation stages of *Bcbva* CI (**A**) illustrated by transmission electron microscopy (TEM) and frequency of sporulation stages at different time points (2, 4, 5, 6, 7, 8, 9 h) during growth of *Bcbva* CI (**B**) and *Ba* 14RA5914 (**C**). Stage 1, vegetative cell; stage 2, lytic cell; stage 3, dividing cell (arrows indicate the infoldings of the cell membrane); stage 4, beginning of engulfment; stage 5, end of engulfment; stage 6, end of engulfment and beginning of spore differentiation; stage 7, differentiated intracellular spore; stage 8, extracellular spore. Bars represent 500 nm.

For a morphological comparison of sporulation in *Ba* 14RA5914 and *Bcbva* CI by thin section electron microscopy, bacterial suspensions were grown in sporulation medium (MGM) and fixed at 1 h intervals (between 2 and 9 h of incubation). Preliminary experiments had shown that all four strains had almost completely sporulated after 24 h, as was shown for *Bcbva* CI (see Section 3.4 and Table S4). Therefore, this time point was omitted for the wild type strains. The sporulation stages from the beginning of engulfment to the extracellular spore are defined and shown in Figure 3 and Supplementary Figure S3. The analysis revealed no apparent differences between the morphology of the sporulation stages of these two strains.

For an overview of the sporulation kinetics, the frequency of the different sporulation stages at the different time points was calculated (Figure 3 and Supplementary Table S5). No signs of sporulation initiation were observed until four hours of growth in both strains. After five hours, engulfment had started in almost 5% of *Bcbva* cells (stage 4), but was not yet visible in *Ba* cells. After six hours, only 34% of *Ba* cells had started engulfment (stage 4), whereas it was already completed (stage 5) in almost 21% of *Bcbva* cells. One and two hours later, the differences between *Ba* and *Bcbva* were less distinct, while after nine hours, spore differentiation had started (stage 6) in 39.4% and had completed (stage 7) in 2.8% of *Ba* cells in contrast to 25.1% and 9% of *Bcbva* cells. No free, extracellular spores were visible after nine hours. A striking difference was the high fraction of lytic cells (stage 2) in *Bcbva* compared to *Ba* between six and nine hours of growth.

### 3.4. Analysis of Two Bcbva CI and CA Sporulation Mutants

After repeated subculturing of the *Bcbva* CI and CA parent strains and selection of phenotypically different colonies, two spontaneous mutants CI-12 and CA-2 were isolated which had apparently lost their sporulation ability. No spores could be detected by malachite green staining and electron microscopy (Supplementary Figure S4), and only vegetative or lytic cells were visible after 24 h.

Cultures of the two mutants were grown for six hours, and heat lysates were tested by PCR for the presence of the intact *sigK* gene or the circularized insertion, as described above, but amplification failed. In contrast, PCRs using primer pairs sigK-for and sigK-3 as well as sigK-4 and sigK-rev were positive, indicating the presence of the *sigK* gene with insertion.

**Figure 4:**
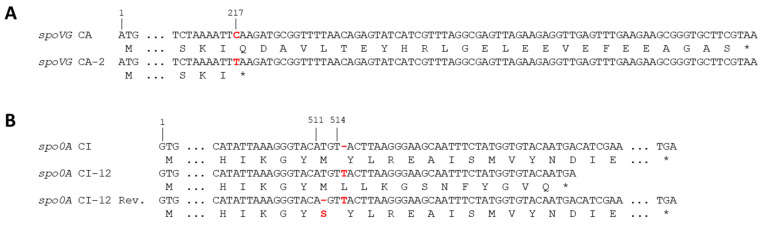
Sequence analysis of the two sporulation mutants. The nonsense mutation at position 217 of the *Bcbva* CA-2 *spoVG* gene is indicated (**A**). The insertion of a T residue behind position 514 of the *Bcbva* CI-12 *spo0A* gene results in a frameshift mutation and a premature stop codon. In the revertants, the deletion of another T residue behind position 511 restores the frame and results in an M → S exchange (M171S) in the Spo0A protein (**B**). The relevant mutations are indicated in red color. *, stop codon; Rev., revertant.

These results pointed to a defect in the sporulation cascade upstream in the activity of the sigma factor σ^K^. Whole genome sequences of the two mutants CI-12 and CA-2 were analyzed for mutations in sporulation-related genes. Indeed, we detected a nonsense mutation in the *spoVG* gene of CA-2 (Figure 4A) and a frameshift mutation (one nucleotide insertion), which resulted in a premature stop codon in the *spo0A* gene of CI-12 (Figure 4B), and these mutations were verified by Sanger sequencing. The corresponding SpoVG and Spo0A proteins of the CA-2 and CI-12 mutants were truncated by 25 and 82 amino acids, respectively.

**Figure 5:**
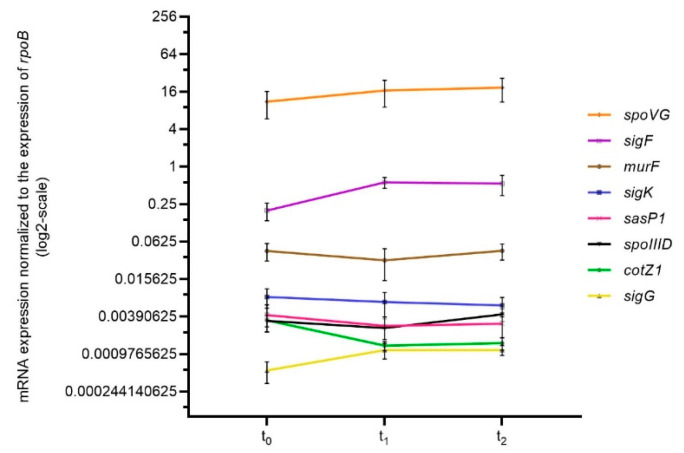
RT-qPCR-based expression profiles of sporulation-associated genes in sporulation mutant *Bcbva* CA-2. Levels of mRNA transcripts were determined for each gene by RNA sampling at time points t_0_ (4 h), t_1_ (5 h) and t_2_ (6 h). For each target gene, relative transcript expression levels were determined by normalization to the control gene *rpoB*. The mean and standard error values from three independent experiments are shown.

As described in Section 3.2, the expression profiles of sporulation-associated genes were determined in *Bcbva* CA-2 to test if genes were expressed despite the mutation in the *spoVG* gene (Figure 5). Gene expression was usually weak and no pronounced up- or down-regulation, as noticed for the wild type strain, was observed at the different time points. One exception was the *spoVG* gene itself, which was still expressed, though not regulated.

To test whether the mutations were reversible, three separate cultures of both mutants were grown in MGM for eight days. The cultures were concentrated 10-fold, but no spores were visible after malachite green staining. After heat inactivation, none of the three cultures of mutant CA-2 showed any growth. In contrast, one of the three cultures of CI-12 showed growth after enrichment with one colony growing on LB agar. This colony as well as two colonies obtained by LB enrichment, here named revertants Rev1, Rev2, and Rev3, were further analyzed. Sanger sequencing of the *spo0A* gene of the three CI-12 revertants revealed the same single nucleotide deletion next to the insertion, suggesting that all revertants can be traced back to the same mutated cell. This intragenic suppressor mutation was able to restore the reading frame of the *spo0A* gene, and the resulting protein contained a serine (polar amino acid) instead of methionine (non-polar amino acid) at position 171 (Figure 4B).

The fraction of heat-resistant spores in the CI wild type and the CI-12 revertant after 24 h growth in MGM was determined. The cultures had sporulated almost completely after one day (Supplementary Table S4), and no differences were visible between CI wild type and CI-12 revertant, which was also confirmed by malachite green staining of spores. Our results show that the M171S amino acid exchange in the Spo0A protein had no influence on the sporulation efficiency.

### 3.5. Further Strains of the B. cereus Group Containing Insertions in the sigK Gene

A database analysis was performed to find further strains possessing an insertion in the *sigK* gene. Interestingly, two apparently identical emetic strains isolated in China [36] and designated *B. paranthracis* EFR-4 (accession number CP064079.1) and *B. cereus* EFR-1 (accession number CP064072.1) contain sequences with high similarity to island IV (Supplementary Figure S5A). The identical insertion sequences of EFR-4 and EFR-1 with a length of 21,367 bp were inserted at exactly the same position of the *sigK* gene as in *Bcbva* and with identical sequences flanking the putative recombination site (Supplementary Figure S5B). Only small regions in the central parts of the insertions are unique for *Bcbva* or EFR-4/EFR-1, respectively. The encoded proteins and their homologies to the proteins of *Bcbva* island IV are listed in Supplementary Table S2. Strikingly, both unique regions encode S subunits of a type I restriction–modification system, with only 30% identity at protein level (55% homology at nucleotide level).

Further strains in the database also possessing insertions in the *sigK* gene do not, however, exhibit homologies to island IV. *B. cereus* AH820 [37]; a periodontal isolate from Norway from 1995 (accession number NC_011773.1) contains an insertion with a size of 38,129 bp (BCAH820_4362 to BCAH820_4417) at position 423 of the *sigK* gene (5′-region: BCAH820_4418; 3′-region: BCAH820_4361). This insertion encodes phage-related proteins and a recombinase (BCAH820_4417) adjacent to the 5′-region of the *sigK* gene. This strain was available, and our PCR analysis and sequencing confirmed the recombination event yielding an intact *sigK* gene and a circularized intervening sequence (Supplementary Figure S5B; data not shown). Two further strains, *B. cereus* J7 (accession number AP022934.1) and *B. cereus* J62 (accession number AP022964.1), both isolated from human blood cultures in Japan in 2006, possess almost identical insertions with a size of 39,036 bp also located at position 423 of the *sigK* gene. The insertions differ from those of *Bcbva* and *B. cereus* AH820 and mainly encode hypothetical proteins. Like *B. subtilis*, these three strains contain the AATGA sequence at the recombination site (Supplementary Figure S5B). All strains possessing insertions in the *sigK* gene belong to the newly proposed *Bacillus mosaicus* genomospecies [38], as shown in the dendrogram in Supplementary Figure S5C.

## 4. Discussion

Efficient sporulation is a prerequisite for the survival of *B. anthracis* after the death of its host and for the initiation of the next infection cycle. Thus, regulation of sporulation initiation and synthesis of the different spore layers are very complex and fine-tuned processes involving a number of various factors. Each interference with this elaborate system might prevent sporulation and result in the loss of viability. Therefore, the identification of a 22 kb insertion termed island IV in the *sigK* gene for the essential sporulation sigma factor σ^K^ in *B. cereus* biovar *anthracis*, a close relative of *Ba* (Figure 1A), was an unexpected finding. A 48 kb insertion in the *sigK* gene was described as the *skin* element in *B. subtilis* more than 30 years ago [24], but was unknown for bacteria of the *B. cereus* group. As for *B. subtilis*, the insertion is excised in a reciprocal site-specific recombination event, which results in an intact *sigK* gene and a circularized intervening sequence (Figure 1A and Supplementary Figure S1). The recombination takes place after the initiation of sporulation, and the circularized molecule was only visible after six, but not yet after four hours of growth (Figure 1B). Based on the homology with the recombinase SpoIVCA of *B. subtilis*, we assume that the recombinase gene of *Bcbva* is also encoded by the *sigK* insertion adjacent to the 5′ fragment of the *sigK* gene. However, no further homologies were detected between the *skin* element and island IV, which are inserted at different positions of the *sigK* gene (Supplementary Figure S5). In contrast to *B. subtilis*, where the insertion seems to encode a prophage, no phage proteins, but several DNA-modifying proteins including a type I restriction–modification system, are encoded by island IV of *Bcbva* (Supplementary Table S2). This type of restriction–modification system is absent in *Ba*. Instead, *Ba* encodes at least three methylation-dependent type IV restriction endonucleases, two of which are also present in *Bcbva*, just as in other *Ba*-related strains of the *B. cereus* group [39].

Based on the observation that *Bcbva* is capable of efficient sporulation, we intended to analyze if the insertion had any influence on the gene regulation cascade and kinetics of sporulation. Thus, the expression profiles of eight sporulation-related genes in two strains of *Ba* and *Bcbva* each were compared. For *Ba,* the well-known laboratory strain Vollum and the “wild” strain 14RA5914 recently isolated from a dead cow were chosen. The two *Bcbva* strains were isolated from chimpanzees in Côte d’Ivoire and Cameroon and were both shown to possess island IV [6,7]. Gene expression was analyzed at time points t_0_ (end of exponential growth), and one (t_1_) and two (t_2_) hours later (Figure 2). As the two *Bcbva* strains were growing slightly faster than the *Ba* strains, these time points differed between the two species (4, 5, and 6 h for *Bcbva*; 5, 6, and 7 h for *Ba*). The expression analysis was complemented by a morphological analysis at the ultrastructural level as well as by quantification of the different morphological sporulation stages.

Although expression profiles differed among the four strains, they usually exhibited similar trends and did not show patterns correlating with species affiliation. However, transcript levels of the eight genes varied among the four strains, with Ba 14RA5914 usually displaying the highest (*spoIIID*, *sigG*, *sigK*, cotZ, *sasP1*) or lowest (*sigF*, *spoVG*) expression rates at t_2_. This might reflect a very efficient response to nutrient-limited conditions, as has been observed for “wild” strains of *Ba* compared to laboratory strains before [40,41].

It is difficult to compare our results to those previously published for *B. subtilis* or *B. anthracis*, because growth conditions and methods for expression analysis are different. Liu et al. [20] and Bergman et al. [21] used microarray analysis and described five distinct waves of gene expression in *Ba* as cells progressed from exponential growth through sporulation. These waves are indicated for the eight genes that we used for expression analysis (Table 1). Despite the different methods applied, our results, based on reverse transcriptase qPCR, showed similar expression patterns. Genes known to be transcribed early during sporulation, like *sigF* and *spoVG*, were expressed at a higher level at t_0_ compared to t_2_, whereas genes known to be active at later stages showed higher expression at t_2_ compared to t_0_. Also, for *sigK*, expression was highest at t_2_ for all strains, indicating that the excision of the insertion in *Bcbva* strains does not delay transcription.

The only gene for which no regulation was observed was *murF* encoding a protein involved in the synthesis of soluble peptidoglycan precursors. The endospore cortex peptidoglycan is synthesized between the two membranes formed by the engulfment of the forespore. Several Mur proteins are required for cell-wall synthesis during growth and not only for cortex synthesis after the initiation of sporulation. In *B. subtilis*, the abundance of these proteins decreased during the early stationary phase, but increased ≥ 2-fold during later stages of sporulation, depending on σ^K^ activation [42]. Our data indicate a weak constitutive expression of the *murF* gene. End of engulfment (stage 5, Figure 3), indicating the final delimitation of the core from the mother cell, was first observed in around 20% of *Ba* and *Bcbva* cells at time point t_2_ (6 h for *Bcbva*, 7 h for *Ba*). Possibly, an upregulation of the *murF* gene becomes visible later, when more cells have entered this stage and more peptidoglycan precursor molecules have to be synthesized, as peptidoglycan formation is only possible at the curved membrane surface of the core [43]. Expression of *murF* was observed in the latest stage of sporulation, as outlined by Liu et al. [20], whereas, it was not listed as an upregulated gene in the study by Bergman et al. [21].

As already mentioned, the two *Bcbva* strains finished exponential growth around one hour before the two *Ba* strains, resulting in time points t_0_ of four hours for *Bcbva* and five hours for *Ba*, respectively. With two strains per species tested, we cannot be sure if this phenomenon is species or strain specific. It would require analyses of additional strains with more replicates. Nevertheless, electron microscopy suggested that the first steps of sporulation progressed more rapidly in *Bcbva* CI compared to *Ba* 14RA5914 (Figure 3). Some 21% of *Bcbva* cells had finished engulfment (stage 5) after six hours (t_2_ for *Bcbva*), whereas about the same fraction (22%) of *Ba* cells had finished engulfment after seven hours (t_2_ for *Ba*) only. It is difficult to correlate the sporulation stages with the gene expression profiles, and it needs to be considered that gene expression starts before morphological changes can be observed. We were most interested in regulation of σ^K^, a sigma factor which is known to be active in the mother cell after the end of engulfment [8]. For all four strains, expression levels of *sigK* were highest at t_2_, but might yet increase at later time points not analyzed here. Accordingly, stage 5 (end of engulfment) most frequently occurred at t_2_ (i.e., after 7 h of sporulation in *Ba* and after 6 h in *Bcbva*). A striking difference between *Bcbva* CI and *Ba* 14RA5914 was the large fraction of lytic cells in *Bcbva*, especially at later time points of sporulation. We have no explanation for this observation. *B. subtilis* possesses two operons, *skf* (sporulation killing factor) and *sdp* (sporulation delaying protein). The proteins produced by these operons are bacteriocins that cause a large reduction in the number of viable *B. subtilis* cells before these are irreversibly committed to sporulation. This enables the population of cells to literally cannibalize their siblings in order to provide food to the cells resistant to the toxins and to delay the one-way process to the formation of the spores [44]. Neither in *Ba* nor in *Bcbva* were homologues of *skf* and *sdp* operons identified, but it cannot be excluded that the lytic cells of *Bcbva* are the result of a similar “cannibalism mechanism” which is based on other, not yet identified genes.

After repeatedly subculturing the *Bcbva* wild type strains CI and CA, two mutants, CI-12 and CA-2, were identified which had lost the ability to sporulate. In nutrient-rich laboratory cultures, the pressure to sporulate is low, and spontaneous mutations in genes involved in sporulation pathways can accumulate [45]. In mutant CI-12, a frameshift mutation resulted in a premature stop codon in the *spo0A* gene and a truncated Spo0A protein of 182 instead of 264 amino acids, in which the DNA binding motif (amino acids 193 and 224) was completely deleted. After 24 h growth in sporulation medium, lytic cells predominated (Supplementary Figure S4). Once a signal to sporulate is produced by nutrient depletion, the bacteria enter a “do or die” program. If the bacteria initiate the sporulation process without successfully executing it, they undergo an alternative dying process [45]. Interestingly, the frameshift mutation, based on a nucleotide deletion, was suppressed by an adjacent nucleotide insertion. In the resulting CI-12 revertant, the missense mutation (M171S) did not affect sporulation efficiency. Notably, the phosphorylation site essential for Spo0A function is present at the aspartic acid residue at position 56.

In mutant CA-2, a nonsense mutation in the *spoVG* gene resulted in a truncated SpoVG protein of 72 instead of 97 amino acids, which completely and irreversibly impeded sporulation. In *B. subtilis*, SpoVG has little effect on spore formation because of the functional redundancy of SpoVG and SpoIIB [31]. For *Ba*, it was shown that deletion of *spoVG* prevented formation of the asymmetric septum, one of the first steps in sporulation, and no heat-resistant spores were observed [22]. Our results confirm that SpoVG is also essential for sporulation of *Bcbva*. Expression analysis of the CA-2 mutant showed that most genes were expressed at low levels and not up- or downregulated as observed for the CA wild type. Only the *spoVG* gene itself, which is activated by Spo0A, was transcribed at higher levels, but not regulated over time.

The NCBI database was screened to detect further strains of the *B. cereus* group which possess an insertion in the *sigK* gene. In fact, five other strains all belonging to the newly proposed genomospecies *Bacillus mosaicus* [38] (Supplementary Figure S5C) were identified. Most strikingly, the two strains *B. paranthracis* EFR-4 and *B. cereus* EFR-1 (probably the same strain due to apparent sequence identity) possessed an insertion at exactly the same position of the *sigK* gene, flanked by the same imperfect 16 bp inverted repeats, and also the insertion sequences themselves were very similar. Our *Bcbva*-specific PCR targets a fragment of island IV [6], but the finding that further strains might possess this insertion indicates that this marker alone cannot be assumed specific for *Bcbva* any longer. The sequence regions unique to *Bcbva* and EFR-4/EFR-1, respectively, encode subunit S of a type I restriction–modification system. This subunit is required for both restriction and modification and is responsible for the recognition of the DNA sequence specific for the system. Thus, the different S subunits point to different recognition sequences. In *B. cereus* AH820 and *B. cereus* J7/J62, different insertions are located at the same position of the *sigK* gene. Similar to *B. subtilis*, these three strains possess the AATGA repeat sequence flanking the inverted repeats and characterizing the recombination site after excision of the insertion. Obviously, this AATGA sequence is not conserved, because *Bcbva* and EFR-4/EFR-1 contain a different, 18 bp imperfectly repeated sequence which allows correct excision of the insertion. As function and necessity of *sigK* insertions in the *B. cereus* group remain unclear, this open question was extended to further spore-forming bacterial species. A 14.6 kbp prophage-like insertion in the *sigK* gene was also found in a number of *Clostridioides difficile* (former *Clostridium difficile*) strains. The element, referred to as *skin*^Cd^, is inserted at a different position of the *sigK* gene and in the opposite orientation compared to *B. subtilis*, but is likewise excised during sporulation and forms a circular molecule [46]. In contrast to *B. subtilis*, the σ^K^ protein of *C. difficile* does not contain the Pro sequence at the N-terminus, which is cleaved off during processing in *B. subtilis* and the *B. cereus* group. Accordingly, no homologue of the corresponding protease SpoIVFB was found in the *C. difficile* genome. Also, strains of *C. difficile* have been described which lacked an insertion in the *sigK* gene and lost the ability to sporulate. In *B. subtilis*, however, deletion of the *skin* element does not lead to a defect in sporulation [26]. Therefore, it has been discussed that in *C. difficile* the regulated excision of the insertion in the *sigK* gene is an essential process that is dispensable in *B. subtilis* because another regulatory mechanism exists through the processing of the precursor protein [46]. We plan to construct island IV deletion mutants to analyze the significance of the insertion for proper sporulation, i.e., why it is dispensable, as in *B. subtilis*, or essential, as in *C. difficile*.

The function of the insertion in the *sigK* gene remains unclear. Restriction–modification systems serve as efficient bacterial defense mechanisms against phage infection. The presence of two types of restriction–modification systems (type I and type IV) in *Bcbva* might strengthen this protective function and could mean an advantage in the *Bcbva* habitat, a highly diverse rainforest ecosystem. Our analyses showed that, despite the presence of an insertion in the gene for the sigma factor σ^K^, *Bcbva* is able to sporulate efficiently and to display sporulation gene expression profiles similar to *Ba*. The excision of the *sigK* insertion does not retard sporulation. On the contrary, sporulation seems to be initiated more rapidly in the two *Bcbva* strains than in the two *Ba* strains, but further studies are required to assess the question if this insight is based on a strain- or a species-specific characteristic.

## 5. Conclusions

Similar to *B. subtilis*, but in contrast to *B. anthracis* and most other strains of the *B. cereus* group, *B. cereus* biovar *anthracis* contains an insertion in the *sigK* gene for the essential sporulation regulator σ^k^. This 22 kb insertion is excised in the mother cell at a late stage of sporulation resulting in an intact *sigK* gene and a circularized molecule. Among other, mainly hypothetical proteins, the insertion encodes a type I restriction–modification system. The presence of the insertion in *Bcbva* does not influence its sporulation kinetics, as was shown by comparing the gene expression of sporulation-associated genes and the morphological sporulation stages between *Bcbva* and *Ba*. The function and possible necessity of an insertion in the *sigK* gene, which was also identified in a few other strains of the *B. cereus* group, are still unclear.

## Data Availability

The data presented in this study are available on request from the corresponding author.

## Supplementary Materials

The following supporting information can be downloaded at: https://www.mdpi.com/article/10.3390/pathogens12121442/s1; Figure S1: Sequence of the *Bcbva sigK* gene before and after excision of the insertion; Figure S2: Growth curves of *Ba* and *Bcbva* strains in sporulation medium MGM; Figure S3: Sporulation stages of *Ba* 14RA5914 illustrated by transmission electron microscopy (TEM); Figure S4: Representative TEM images of sections from *Bcbva* CI-12 and CA-2 grown for 2 or 24 h in sporulation medium MGM; Figure S5: Strains possessing insertions in the *sigK* gene and comparison of corresponding sequences; Table S1: Primer sequences; Table S2: Open reading frames in the insertion of *Bcbva* CI and their homologies with the related insertion of *B*. *paranthracis* EFR-4 and *B*. *cereus* EFR-1; Table S3: Amplicon sizes and PCR efficiencies of the PCRs used for expression analysis; Table S4: CFU of heat-inactivated (65 °C, 30 min) and untreated cultures of *Bcbva* CI wildtype and CI-12 revertant after 24 h in MGM; Table S5: Quantification of frequency of sporulation stages.
