# Supplementary material for: Analysis of Sporulation in Bacillus cereus Biovar anthracis Which Contains an Insertion in the Gene for the Sporulation Factor σK
Source: Pathogens. 2023 Dec 13;12(12):1442. doi: 10.3390/pathogens12121442 (PMC10745906; doi:10.3390/pathogens12121442)
Supplement: Supplementary file 1 [file pathogens-12-01442-s001.zip › pathogens-2731426-supplementary.pdf]

Analysis of sporulation in *Bacillus cereus* biovar *anthracis* which contains an insertion in the gene for the sporulation factor σ^K

Supplementary Materials

Bcbva sigK gene with insertion (22925 bp):

TTGAGTCTATTCGCCGCAATTGGATATATGGTTCGAGAAGTGTTTGTCTTTGTTTCTTATGTGAAGAACAATGCGTTTCCGCAGCCATTATCAT
CAGACGATGAGAGAAAGTACTTAGAGTTAATGGAGCAAGGTGATGCTCAAGCGAGGAATCTGTTAATTGAACATAATTTACGGCTTGTAGCTCA
TATC**GTTAAG**AGATTGTGTACTCAAAACGATGTCATGGACAAGGGTATCTAATGTACCATTTATTTTAATATTCACTTGCTCTTCCCCATCAAA
CTCTAAATGAATTTCATCAATTAGCTGAGTAATAATATTTCTTTTTTCTGCAAAGGTTAATTTATTATCAGTCTTATCTTTTAATACATTTTGA
ATGGATTCAATAATTTGATCCATTCTTTTTTTTGTGTTTTCCTTTTTGTTTTCTAAATTTATTTT**GTCTTGGAATGCTTGTAATTCT**TCTTTTA
ATTTACTTATTTGTTCATTTATT_21479bp_TTTTTTCTATCTCAGTCTCATCATATATTC**TTCTAACTAATCGATAATGCGC**TCCCATTAAT
TTTCTCCCCCTAGTATATATTTAAGTCATTTACTTGCTAATTATTCAATTATCTCAAAAAAGGTTTTTTTTGGATACAATTTCTTTATTTGTTA
ATGGAAATAAAAAAACGAGCGATTTAAAAATAGCTTATTTATGTAGTCCAGAACCTCAAAATGATTAAGTAGGAATAAATTTTCGGTATCCCCC
CACTAAAGGTGTGTTACCTTTAGTGGGGGTAATTGAAGTTTGTCCGATTTTAATAAAGATATAATTTGTAATTTTTTTGATCAATTATCTTTTT
CTGTTGCTTTGTATTTCTTACCAGTGTAAAACCAGCATAAATTAGGATGTTAATAAGGATATTTTGACGTTCTTTTGTGTTCATAAC**GTCAAA**A
AATTTGAAAATACAGGGGAAGATGCAGAAGATTTAATTTCAATTGGTACAATCGGGCTCATTAAAGCGATTGAGAGCTATTCGGCAGGAAAAGG
TACAAAACTTGCGACGTACGCAGCACGCTGTATTGAAAATGAAATTTTGATGCATTTACGTGTATTAAAGAAAACGAAAAAGGACGTTTCACTT
CATGATCCAATCGGGCAAGATAAAGAGGGGAATGAAATATCGCTTATTGATATATTAAAATCAGAGTCTGAAGATGTAATTGATATGATCCAGC
TTAGTATGGAGTTAGAAAAGATTAAAGAGTATATCGATATTTTAGACGAACGAGAGAAAGAAGTAATCGTGAAGCGTTTTGGACTGGGGCTTGA
TAAGGAGAAAACGCAACGAGAGATTGCGAAGGCACTTGGTATTTCCAGAAGCTATGTATCAAGAATTGAAAAGCGCGCTTTAATGAAAATGTTC
CATGAATTTGTAAGA**GCAGAGAAAGAGAAAAAAGCAAAAGAATAA**

Intact *sigK* gene after excision of insertion (714 bp):

TTGAGTCTATTCGCCGCAATTGGATATATGGTTCGAGAAGTGTTTGTCTTTGTTTCTTATGTGAAGAACAATGCGTTTCCGCAGCCATTATCAT
CAGACGATGAGAGAAAGTACTTAGAGTTAATGGAGCAAGGTGATGCTCAAGCGAGGAATCTGTTAATTGAACATAATTTACGGCTTGTAGCTCA
TATC**GTTAAA**AAATTTGAAAATACAGGGGAAGATGCAGAAGATTTAATTTCAATTGGTACAATCGGGCTCATTAAAGCGATTGAGAGCTATTCG
GCAGGAAAAGGTACAAAACTTGCGACGTACGCAGCACGCTGTATTGAAAATGAAATTTTGATGCATTTACGTGTATTAAAGAAAACGAAAAAGG
ACGTTTCACTTCATGATCCAATCGGGCAAGATAAAGAGGGGAATGAAATATCGCTTATTGATATATTAAAATCAGAGTCTGAAGATGTAATTGA
TATGATCCAGCTTAGTATGGAGTTAGAAAAGATTAAAGAGTATATCGATATTTTAGACGAACGAGAGAAAGAAGTAATCGTGAAGCGTTTTGGA
CTGGGGCTTGATAAGGAGAAAACGCAACGAGAGATTGCGAAGGCACTTGGTATTTCCAGAAGCTATGTATCAAGAATTGAAAAGCGCGCTTTAA
TGAAAATGTTCCATGAATTTGTAAGA**GCAGAGAAAGAGAAAAAAGCAAAAGAATAA**

Circularized insertion after excision (22211 bp):

21479bp_AATAAATGAACAAATAAGTAAATTAAAAGA**AGAATTACAAGCATTCCAAGAC**AAAATAAATTTAGAAAACAAAAAGGAAAACACAA
AAAAAAGAATGGATCAAATTATTGAATCCATTCAAAATGTATTAAAAGATAAGACTGATAATAAATTAACCTTTGCAGAAAAAAGAAATATTAT
TACTCAGCTAATTGATGAAATTCATTTAGAGTTTGATGGGGAAGAGCAAGTGAATATTAAAATAAATGGTACATTAGATACCCTTGTCCATGAC
ATCGTTTTGAGTACACAATCT**CTTGAC**GTTATGAACACAAAAGAACGTCAAAATATCCTTATTAACATCCTAATTTATGCTGGTTTTACACTGG
TAAGAAATACAAAGCAACAGAAAAAGATAATTGATCAAAAAAATTACAAATTATATCTTTATTAAAATCGGACAAACTTCAATTACCCCCACTA
AAGGTAACACACCTTTAGTGGGGGGATACCGAAAATTTATTCCTACTTAATCATTTTGAGGTTCTGGACTACATAAATAAGCTATTTTTAAATC
GCTCGTTTTTTTATTTCCATTAACAAATAAAGAAATTGTATCCAAAAAAAACCTTTTTTGAGATAATTGAATAATTAGCAAGTAAATGACTTAA
ATATATACTAGGGGGAGAAAATTAATGGGA**GCGCATTATCGATTAGTTAGAA**GAATATATGATGAGACTGAGATAGAAAAAA

Figure S1. Sequence of the *Bcbva sigK* gene before and after excision of the insertion. Primers sigK-for and sigK-rev are indicated in bold, primers sigK-3 and sigK-4 in blue, and bases at the recombination site in red and green. The 16-bp imperfect inverted repeats are underlined.

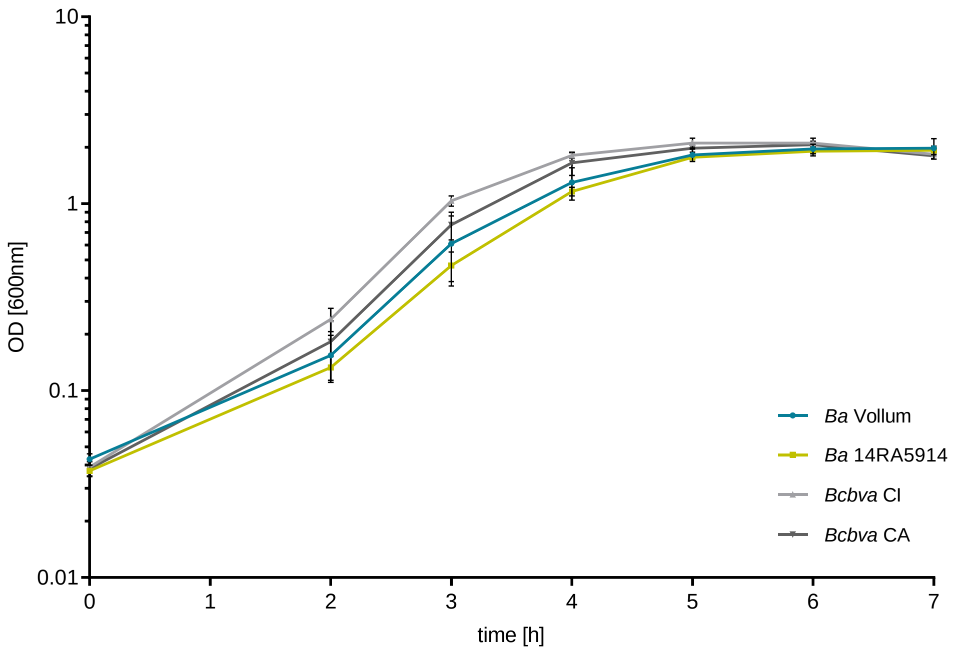

Figure S2. Growth curves of *Ba* and *Bcbva* strains in sporulation medium MGM. Averages and standard errors based on 3 independent replicates were calculated using the GraphPad Prism 9 Software.

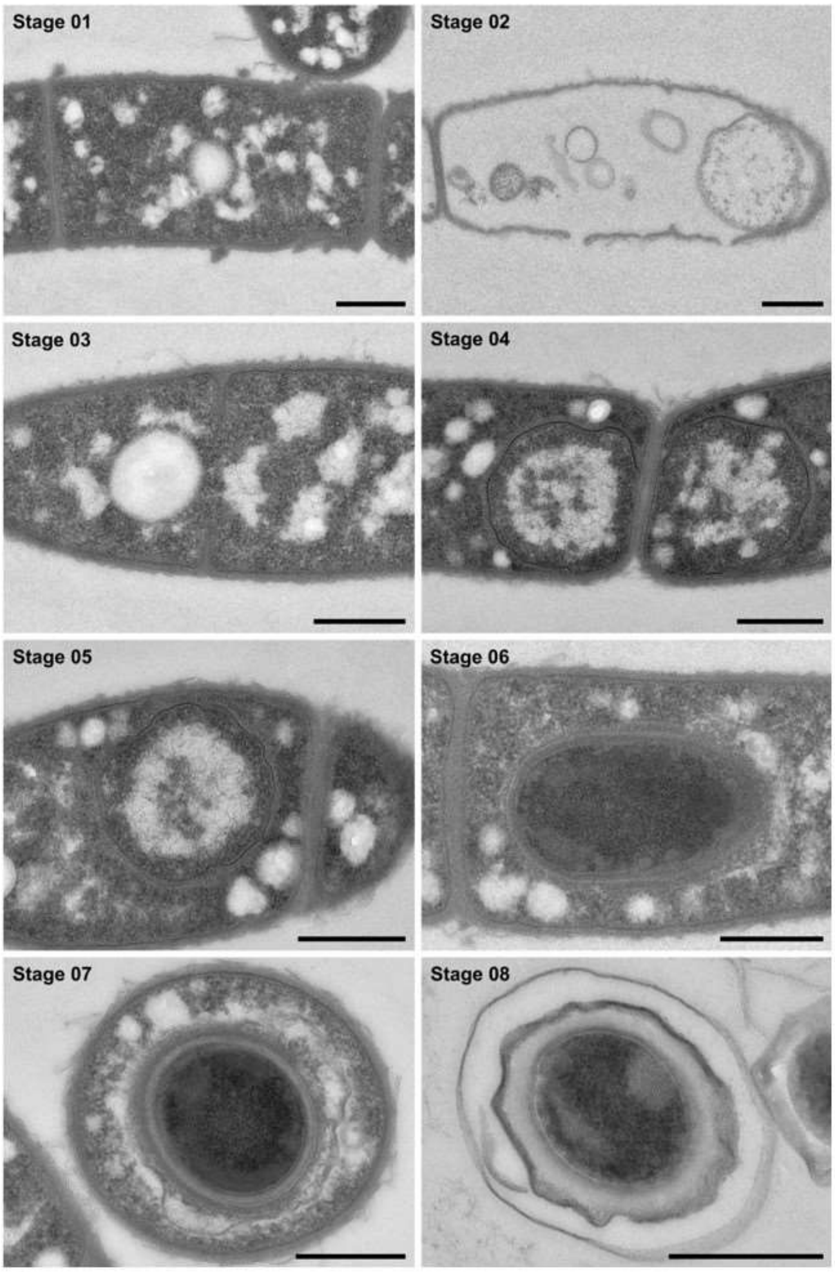

Figure S3. Sporulation stages of *Ba* 14RA5914 illustrated by transmission electron microscopy (TEM). Stage 1, vegetative cell; stage 2, lytic cell; stage 3, dividing cell; stage 4, beginning of engulfment; stage 5, end of engulfment; stage 6, end of engulfment and beginning of spore differentiation; stage 7, differentiated intracellular spore; stage 8, extracellular spore. Bars represent 500 nm.

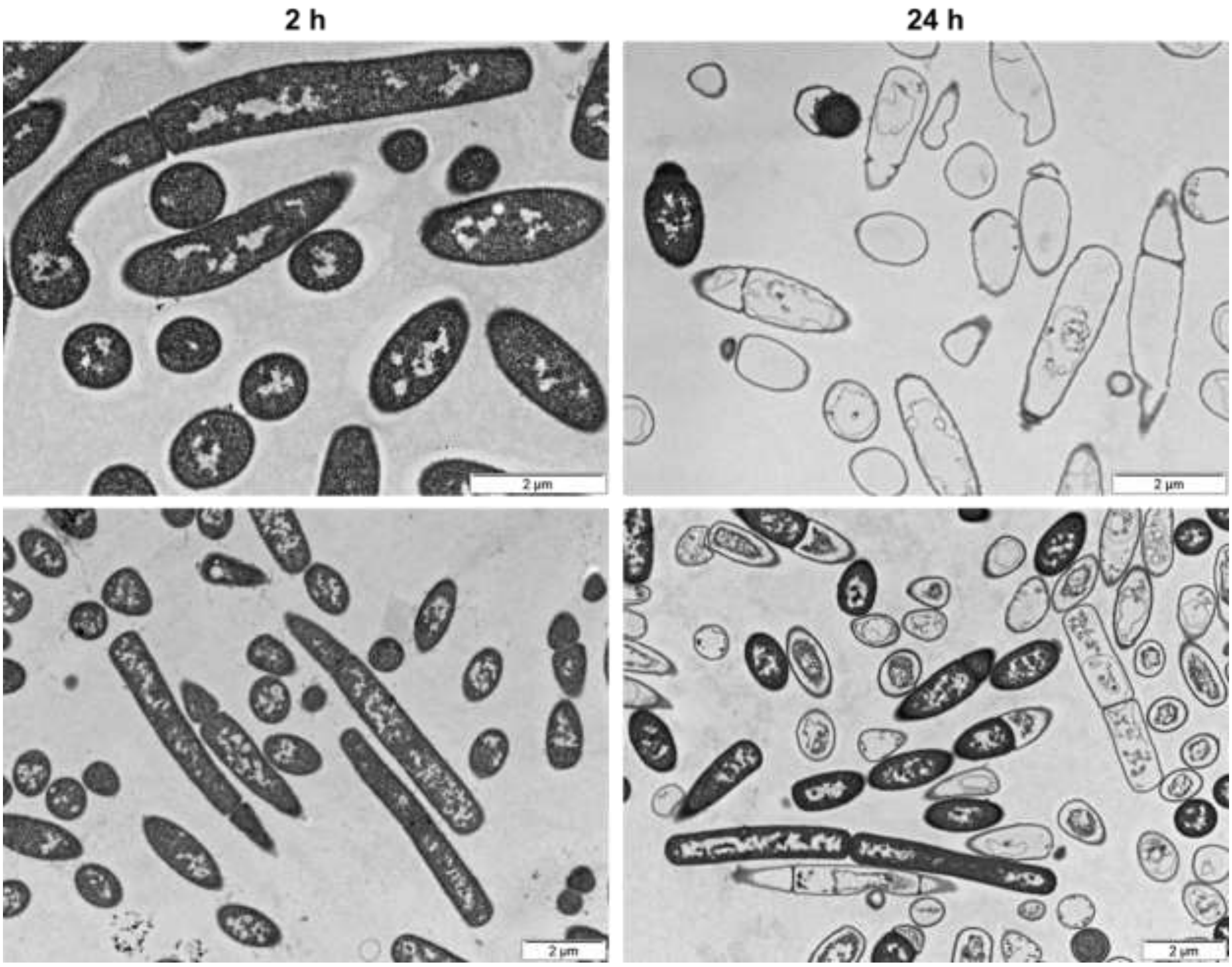

Figure S4. Representative TEM images of sections from *Bcbva* CI-12 (upper panel) and CA-2 (lower panel) grown for 2 or 24 hours in sporulation medium MGM. At 2 h of incubation in MGM bacteria reveal the typical ultrastructure of vegetative bacteria. After 24 h of incubation in MGM most cells appear extracted or show a condensed cytoplasm, but no spores or stages of sporulation.

A

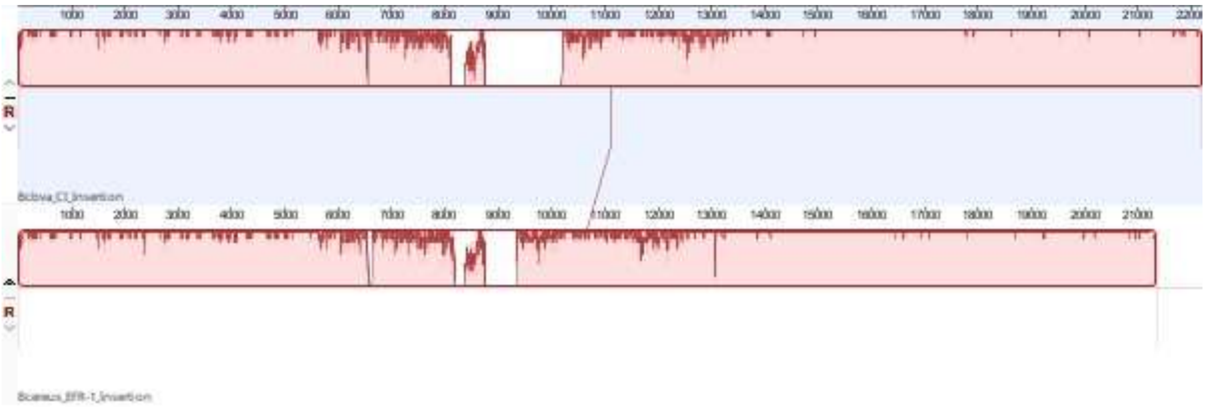

B

B. cereus biovar *anthracis*

sigK, 5’: TTG ... AACATAATTTACGGCTTGTAGCTCATATCGTTAAGAGATTGTGTACTCAAAACGATGTCATGGA ...
sigK, 3’: ... ATATTTTGACGTTCTTTTGTGTTCATAACGTCAAAAAATTTGAAAATACAGGGGAAGATGCAGA ... TAA
intact gene: TTG ... AACATAATTTACGGCTTGTAGCTCATATCGTTAAAAAATTTGAAAATACAGGGGAAGATGCAGA ... TAA

B. paranthracis EFR-4 and *B. cereus* EFR-1

sigK, 5’: TTG ... AACATAATTTACGGCTTGTAGCTCATATCGTTAAGAGATTGTGTACTCAAAACGATGTCATGGG ...
sigK, 3’: ... ATATTTTGACGTTCTTTTGTGTTCATAACGTCAAAAAATTTGAAAATACAGGGGAAGATGCAGA ... TAA
intact gene: TTG ... AACATAATTTACGGCTTGTAGCTCATATCGTTAAAAAATTTGAAAATACAGGGGAAGATGCAGA ... TAA

B. cereus AH820

sigK, 5’: TTG ... ATGATCCAATCGGGCAAGATAAAGAGGGG**AATGA**GATGCCGACTAGACTTTATCCTGTTTAGAAA ...
sigK, 3’: ... TGAATAAAACAGGATAAAGTCTAGTGGGG**AATGA**AATATCGCTTATTGATATATTAAAATCAGAG ... TAA
intact gene: TTG ... ATGATCCAATCGGGCAAGATAAAGAGGGG**AATGA**AATATCGCTTATTGATATATTAAAATCAGAG ... TAA

B. cereus J7 and *B. cereus* J62

sigK, 5’: TTG ... ATGATCCAATCGGGCAAGATAAAGAGGGG**AATGA**GTTACCAACTAGACTTTATCCTGTTTCTTAT ...
sigK, 3’: ... GAGATACAACAGGATAAAGTCTAGTGGGG**AATGA**AATATCGCTTATTGATATATTAAAATCAGAG ... TAA
intact gene: TTG ... ATGATCCAATCGGGCAAGATAAAGAGGGG**AATGA**AATATCGCTTATTGATATATTAAAATCAGAG ... TAA

B. subtilis

sigK, 5’: GTG ... GCGACGTATGCAGCGAGGTGTATTGAA**AATGA**GATTGTAATTACAAAAGGGGGGTGCATACA ...
sigK, 3’: ... TTTATGCCCCCCCTTTTTGTAATGAAT**AATGA**AATCCTCATGCATTTGCGCGCATTGAAAAA ... TAA
intact gene: GTG ... GCGACGTATGCAGCGAGGTGTATTGAA**AATGA**AATCCTCATGCATTTGCGCGCATTGAAAAA ... TAA

C

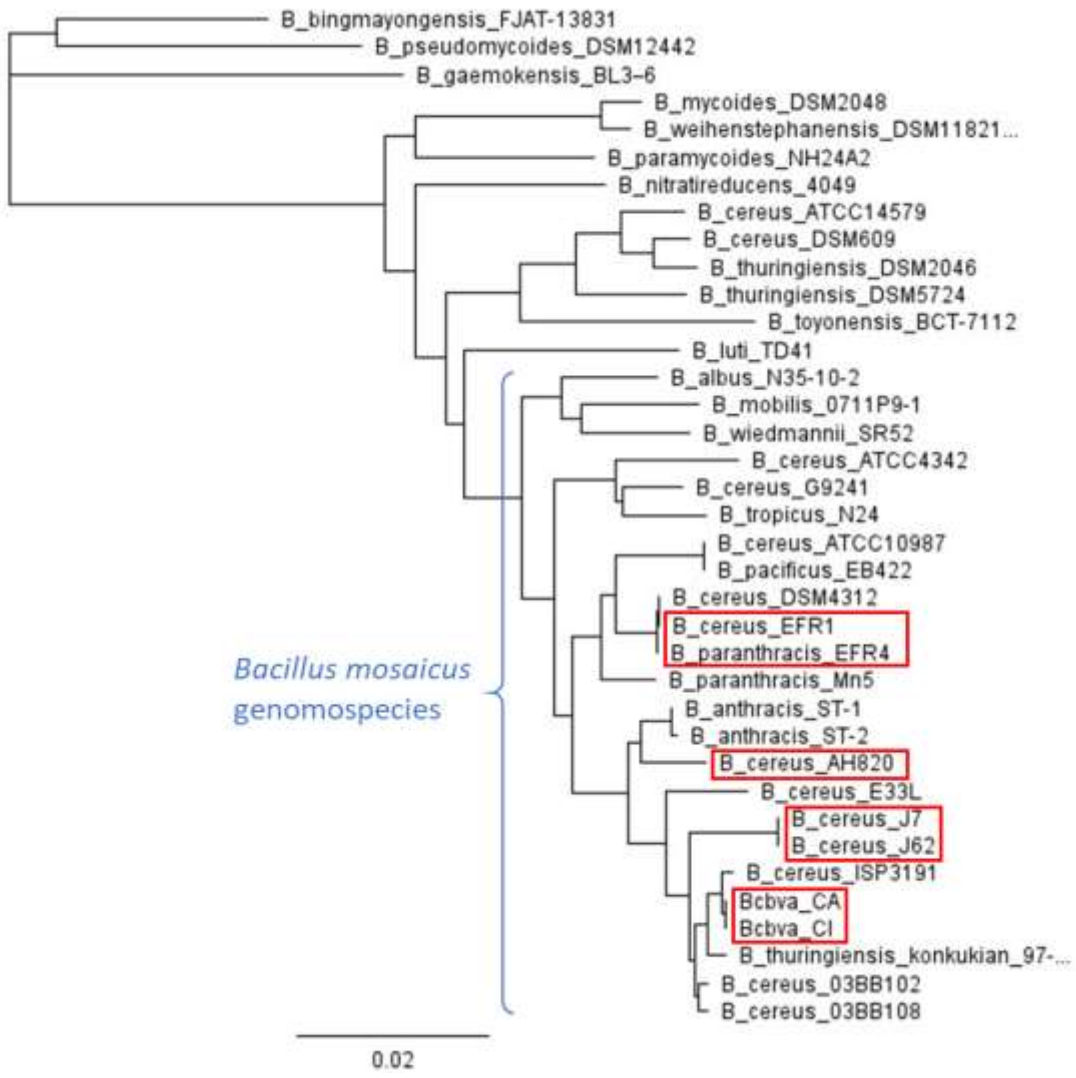

Figure S5. Strains possessing insertions in the *sigK* gene and comparison of corresponding sequences. A: Mauve alignment (created in Geneious version 2021.2) of the *sigK* insertions of *Bcbva* CI (upper graph) and *B. cereus* EFR-1 (lower graph) indicating homologous and unique sequences.

B: Comparison of the insertion sites in *Bcbva*, further strains of the *B. cereus* group, and *B. subtilis*. A 18 bp imperfectly repeated sequence within which the recombination takes place in *Bcbva* and *B. paranthracis* EFR4/*B. cereus* EFR1 is highlighted in grey. The AATGA sequence at the recombination site (if present) is indicated in bold, inverted repeats are underlined. C: Neighbor-Joining tree (created in Geneious version 2021.2) based on the concatenated sequences of the seven housekeeping genes *glp*, *gmk*, *ilv*, *pta*, *pur*, *pyc*, *tpi* (https://pubmlst.org/organisms/bacillus-cereus). Strains containing insertions in the *sigK* gene are members of the *Bacillus mosaicus* genomospecies and belong to sequence types ST 26 (*B. cereus* EFR-1 and *B. paranthracis* EFR-4), ST 460 (*B. cereus* AH820), ST 167 (*B. cereus* J7 and J62) and ST 935 (*Bcbva* CI and CA).

Table S1. Primer sequences.

Primer	Sequences in 5‘-3‘ direction
rpoB-for	CGGCAGCGACAGCTTGTATT
rpoB-rev	AACCGCCTGACGTTGCA
sigK-for	TTGAGTCTATTCGCCGCAATTGG
sigK-rev	TTATTCTTTTGCTTTTTTCTCTTTCTCTGC
sigK-3	AGAATTACAAGCATTCCAAGAC
sigK-4	TTCTAACTAATCGATAATGCGC
sigK-rev2*	TCATCGTCTGATGATAATGGC
sigG-for	GCAAAGTGGAGAGATAAGCG
sigG-rev	CATAAGTCCGATACAACCAAC
spoIIID-for	GTGCACGATTACATCAAAGAG
spoIIID-rev	GTTCTGTTAAATCTTTATGGACTG
spoVG-for	GCCTCTATTACTCTAGACCATGAA
spoVG-rev	GTGCAATGTCACGGAATTCTCC
cotZ-for	CACCATGGCTCTTCTCATTG
cotZ-rev	CTGATGCAGTATTGTGTGCG
sigF-for	AGAAACCTCAGTTAAAGGACC
sigF-rev	TGTACAACCGACCATACGAG
sasP-for	ACCAGGAGCAACAGCTGCAA
sasP-rev	ACCAGGAGCAACAGCTGCAA
murF-for	GGAATGTCAAGCCGCGGAGA
murF-rev	GCCTCACGAGAGCCTAAGTCC

* reverse primer for expression analysis

Table S2. Open reading frames in the insertion of *Bcbva* CI and their homologies with the related insertion of *B. paranthracis* EFR-4 and *B. cereus* EFR-1.

**Locus Tag in *Bcbva* CI**	**Annotation**	**Protein size in *Bcbva* CI**	**Locus Tag and protein size in *B. paranthracis* EFR-4 / *B. cereus* EFR-1**	**Identity at protein level**
not annotated	*sigK* gene, 5'-end (encoding 65 aa up to recombination site)	79 aa	INR14_21740 / INQ58_21740, 79 aa	78/79 aa, 99% (first 65 aa identical)
BACI_c43240	recombinase family protein	545 aa	INR14_21735 / INQ58_21735, 545 aa	534/545 aa, 98%
BACI_c43230	helix-turn-helix domain-containing protein	148 aa	INR14_21730 / INQ58_21730, 148 aa	145/148 aa, 98%
BACI_c43220	hypothetical protein	160 aa	INR14_21725 / INQ58_21725, 169 aa	157/160 aa, 98%
BACI_c43210	recombinase family protein	532 aa	INR14_21720 / INQ58_21720, 532 aa	524/532 aa, 98%
BACI_c43200	hypothetical protein	57 aa	INR14_21715 / INQ58_21715, 57 aa	100%
BACI_c43190	helix-turn-helix transcriptional regulator	70 aa	INR14_21710 / INQ58_21710, 70 aa	100%
BACI_c43180	hypothetical protein	124 aa	INR14_21705 / INQ58_21705, 124 aa	120/124 aa, 97%
BACI_c43170	putative endonuclease	182 aa	INR14_21700 / INQ58_21700, 182 aa	170/182 aa, 94%
BACI_c43160	type I restriction-modification system subunit M*	484 aa	INR14_21695 / INQ58_21695, 484 aa	437/466 aa, 94%
BACI_c43150	type I restriction modification enzyme subunit S	369 aa	INR14_21690 / INQ58_21690, 382 aa	98/328 aa, 30%
BACI_c43140	hypothetical protein	222 aa	absent	
BACI_c43130	type I restriction-modification endonuclease, subunit R*	1113 aa	INR14_21685 / INQ58_21685, 1113 aa	1075/1113 aa, 97%
BACI_c43120	hypothetical protein	83 aa	INR14_21680 / INQ58_21680, 83 aa	100%
BACI_c43110	dsDNA nuclease domain-containing protein*	616 aa	INR14_21675 / INQ58_21675, 616 aa	100%
BACI_c43100	hypothetical protein	41 aa	INR14_21670 / INQ58_21670, 203 aa	100%
BACI_c43090	SMC family ATPase*	1001 aa	INR14_21665 / INQ58_21665, 1001 aa	1000/1001 aa, 99%
BACI_c43080	hypothetical protein	265 aa	INR14_21660 / INQ58_21660, 273 aa	263/265 aa, 99%
BACI_c43070	sigma factor σ^K (*sigK* gene, 3'-end)	181 aa	INR14_21655 / INQ58_21655, 181 aa	100%

aa, amino acids

*frequent genes, also present in other strains of the *B. cereus* group

Table S3. Amplicon sizes and PCR efficiencies of the PCRs used for expression analysis.

PCR assay	Amplicon size (bp)	PCR efficiency			
		Ba Vollum	*Ba* 14RA5914	*Bcbva* CI	*Bcbva* CA
sigK	104	1.9420	1.9396	1.9503	1.9195
sigG	136	1.9200	1.9685	1.9530	1.9605
spoIIID	130	1.9130	2.0440	1.9495	1.9490
spoVG	124	1.9698	1.9430	1.9796	1.9460
cotZ	127	1.9286	1.9651	1.9640	1.9451
sigF	121	1.9475	1.9833	1.9575	1.9599
sasP	112	1.9252	1.9416	1.9584	1.9419
murF	113	1.9215	1.9489	1.9517	1.9491
rpoB	86	1.9380	1.9448	1.9383	1.9370

Table S4. Colony forming units of heat-inactivated (65°C, 30 min) and untreated cultures of *Bcbva* CI wildtype and CI-12 revertant after 24 hours in MGM. Three replicates of each strain were tested.

Strain		cfu/ml	sporulation efficiency
Bcbva CI, rep. 1	untreated	1.6×10^8	100%
	heat	2.1×10^8	
Bcbva CI, rep. 2	untreated	7.9×10^7	100%
	heat	1.6×10^8	
Bcbva CI, rep. 3	untreated	1.5×10^8	93.3%
	heat	1.4×10^8	
Bcbva CI-12 revertant, rep. 1	untreated	7.6×10^7	93.4%
	heat	7.1×10^7	
Bcbva CI-12 revertant, rep. 2	untreated	1.1×10^8	84.5%
	heat	9.3×10^7	
Bcbva CI-12 revertant, rep. 3	untreated	7.8×10^7	100%
	heat	8.7×10^7	

rep., replicate
cfu, colony forming units

Table S5. Quantification of frequency of sporulation stages.

Growth [h]	Number/percentage of *Ba* 14RA5914 cells in stage								
	**1**	**2**	**3**	**4**	**5**	**6**	**7**	**8**	**Total**
**2**	193	1	6	0	0	0	1	0	201
	96.0	0.5	3.0	0.0	0.0	0.0	0.5	0.0	100
**4**	141	1	10	0	0	0	0	0	152
	92.8	0.5	6.8	0.0	0.0	0.0	0.0	0.0	100
**5**	201	2	11	0	0	0	0	0	214
**(t_0)**	93.9	0.9	5.1	0.0	0.0	0.0	0.0	0.0	100
**6**	156	1	12	88	1	0	0	0	258
**(t_1)**	60.5	0.4	4.7	34.1	0.4	0.0	0.0	0.0	100
**7**	116	9	6	11	47	28	0	0	217
**(t_2)**	53.5	4.1	2.8	5.1	21.7	12.9	0.0	0.0	100
**8**	99	13	5	4	54	54	1	0	230
	43.0	5.7	2.2	1.7	23.5	23.5	0.4	0.0	100
**9**	98	20	4	0	20	97	7	0	246
	39.8	8.1	1.6	0.0	8.1	39.4	2.8	0.0	100
**Growth [h]**	**Number/percentage of *Bcbva* CI cells in stage**								
	**1**	**2**	**3**	**4**	**5**	**6**	**7**	**8**	**Total**
**2**	234	1	8	0	0	0	0	0	243
	96.3	0.4	3.3	0.0	0.0	0.0	0.0	0.0	100
**4**	246	1	6	0	0	0	0	0	253
**(t_0)**	97.2	0.4	2.4	0.0	0.0	0.0	0.0	0.0	100
**5**	231	20	8	13	0	0	0	0	272
**(t_1)**	84.9	7.4	2.9	4.8	0.0	0.0	0.0	0.0	100
**6**	120	48	1	14	50	7	0	0	240
**(t_2)**	50.0	20.0	0.4	5.8	20.8	2.9	0.0	0.0	100
**7**	85	61	4	0	58	30	0	0	238
	35.7	25.6	1.7	0.0	24.4	12.6	0.0	0.0	100
**8**	96	57	1	0	31	54	2	0	241
	39.8	23.7	0.4	0.0	12.9	22.4	0.8	0.0	100
**9**	102	61	1	0	4	64	23	0	255
	40.0	23.9	0.4	0.0	1.6	25.1	9.0	0.0	100
